# The Contrasting Effect of Macromolecular Crowding on Amyloid Fibril Formation

**DOI:** 10.1371/journal.pone.0036288

**Published:** 2012-04-30

**Authors:** Qian Ma, Jun-Bao Fan, Zheng Zhou, Bing-Rui Zhou, Sheng-Rong Meng, Ji-Ying Hu, Jie Chen, Yi Liang

**Affiliations:** State Key Laboratory of Virology, College of Life Sciences, Wuhan University, Wuhan, China; University of Maryland, United States of America

## Abstract

**Background:**

Amyloid fibrils associated with neurodegenerative diseases can be considered biologically relevant failures of cellular quality control mechanisms. It is known that *in vivo* human Tau protein, human prion protein, and human copper, zinc superoxide dismutase (SOD1) have the tendency to form fibril deposits in a variety of tissues and they are associated with different neurodegenerative diseases, while rabbit prion protein and hen egg white lysozyme do not readily form fibrils and are unlikely to cause neurodegenerative diseases. In this study, we have investigated the contrasting effect of macromolecular crowding on fibril formation of different proteins.

**Methodology/Principal Findings:**

As revealed by assays based on thioflavin T binding and turbidity, human Tau fragments, when phosphorylated by glycogen synthase kinase-3β, do not form filaments in the absence of a crowding agent but do form fibrils in the presence of a crowding agent, and the presence of a strong crowding agent dramatically promotes amyloid fibril formation of human prion protein and its two pathogenic mutants E196K and D178N. Such an enhancing effect of macromolecular crowding on fibril formation is also observed for a pathological human SOD1 mutant A4V. On the other hand, rabbit prion protein and hen lysozyme do not form amyloid fibrils when a crowding agent at 300 g/l is used but do form fibrils in the absence of a crowding agent. Furthermore, aggregation of these two proteins is remarkably inhibited by Ficoll 70 and dextran 70 at 200 g/l.

**Conclusions/Significance:**

We suggest that proteins associated with neurodegenerative diseases are more likely to form amyloid fibrils under crowded conditions than in dilute solutions. By contrast, some of the proteins that are not neurodegenerative disease-associated are unlikely to misfold in crowded physiological environments. A possible explanation for the contrasting effect of macromolecular crowding on these two sets of proteins (amyloidogenic proteins and non-amyloidogenic proteins) has been proposed.

## Introduction

Amyloid fibril formation has been traditionally and widely investigated in dilute solutions [1]–[4]. However, the inside of a cell is poorly modeled by such dilute solutions and biochemical reactions within cells including fibril formation differ greatly from those in dilute solutions [5]–[15]. One of the differences is that most biological fluids contain a high total concentration of macromolecules, termed macromolecular crowding or crowded physiological environments [6]–[8].

Amyloid fibrils associated with neurodegenerative diseases such as Alzheimer disease, prion disease, and amyotrophic lateral sclerosis (ALS) [1], [16]–[18] can be considered biologically relevant failures of cellular quality control mechanisms including molecular chaperones, proteolysis, autophagy, and proteasomes [19], [20]. Human Tau protein forms filaments in the brains of patients with Alzheimer disease [17], and glycogen synthase kinase-3β (GSK-3β) phosphorylation plays an important role in Alzheimer disease [11]. It is known that *in vivo* human Tau protein [17], [20], human prion protein (PrP) and its pathogenic mutants [16], [21], [22], and human copper, zinc superoxide dismutase (SOD1) pathogenic mutants [18], [23], [24] have the tendency to form fibril deposits in a variety of tissues and they are associated with Alzheimer disease, prion disease, and ALS, respectively, while the rabbit PrP [13], [25], [26] and hen egg white lysozyme [27] do not readily form fibrils and are unlikely to cause neurodegenerative diseases. Furthermore, misfolded Tau protein accumulating in Alzheimer disease and misfolded SOD1 accumulating in ALS can cause aggregation of their native counterparts in crowded physiological environments through a mechanism similar to the infectious prion protein PrP^Sc^ causing aggregation of its cellular isoform PrP^C^ [18], [20].

In this study, we want to know the role of crowded physiological environments in amyloid fibril formation. We investigated the contrasting effect of macromolecular crowding on fibril formation of amyloidogenic proteins, such as GSK-3β phosphorylated human Tau protein, human PrP and its pathogenic mutants E196K and D178N, and pathological human SOD1 mutant A4V, and non-amyloidogenic proteins, such as the rabbit PrP and hen egg white lysozyme, by using thioflavin T (ThT) binding and turbidity assays. We demonstrated that macromolecular crowding dramatically promoted fibril formation of these amyloidogenic proteins but remarkably inhibited aggregation of the two non-amyloidogenic proteins. Our results suggest that proteins associated with neurodegenerative diseases are more likely to form amyloid fibrils in crowded physiological environments than in dilute solutions but some non-amyloidogenic proteins are unlikely to aggregate and to form amyloid fibrils in crowded physiological environments.

## Materials and Methods

### Ethics statement

All research involving original human work was approved by the Institutional Review Board of the College of Life Sciences, Wuhan University (Wuhan, China), leaded by Dr. Hong-Bing Shu, the Dean of the college, in accordance with the guidelines for the protection of human subjects. Written informed consent for the original human work that produced the plasmid samples was obtained.

### Materials

The crowding agents, Ficoll 70, Ficoll 400, dextran 70, polyethylene glycol (PEG) 2000, and PEG 20000 were purchased from Sigma-Aldrich (St. Louis, MO). Heparin (average MW = 6 kDa) and ThT were also obtained from Sigma-Aldrich. Dithiothreitol (DTT), urea, and Sarkosyl were purchased from Amresco (Solon, OH). Guanidine hydrochloride (GdnHCl) was obtained from Promega (Madison, WI). All other chemicals used were made in China and were of analytical grade.

### Plasmids and proteins

The cDNA encoding human Tau fragments Tau_244–372_ and Tau_244–441_ were amplified using the plasmid for human Tau40 (kindly provided by Dr. Michel Goedert) as a template. The PCR-amplified fragments were subcloned into pRK172 vector. Recombinant Tau_244–372_ and Tau_244–441_ were expressed in *Escherichia coli* and purified to homogeneity by SP-Sepharose chromatography as described [2], [28]. Purified Tau protein was analyzed by SDS-PAGE with one band and confirmed by mass spectrometry. The concentrations of human Tau fragments were determined according to their absorbance at 214 nm with a standard curve drawn by bovine serum albumin. His-tagged GSK-3β cDNA was amplified using human GSK-3β plasmid (kindly provided by Dr. Thilo Hagen) as a template. Recombinant GSK-3β was expressed in *Escherichia coli* and purified to homogeneity by Ni-NTA-Sepharose and SP-Sepharose chromatography sequentially as described [11].

The human/rabbit PrP cDNA was subcloned into pET30a vector. Single mutants of human PrP were generated using primers ACAACCACCAAGGGGAAGAACTTCA CCGAG/CTCGGTGAAGTTCTTCCCCTTGGTGGTTGT for E196K and AACAACTTTGTGCACAACTGCGTCAATATCAC/GTGATATTGACGCAGTTGTGCACAAAGTTGTT for D178N. Recombinant full-length human/rabbit prion proteins and two pathogenic human PrP mutants E196K and D178N were expressed in *Escherichia coli*, isolated on a Ni-Sepharose column, and further purified by HPLC on a C4 reversed-phase column (Shimadzu, Kyoto, Japan) as described by Bocharova and co-workers [29]. Purified human/rabbit prion proteins were confirmed by SDS-PAGE and mass spectrometry to be single species with an intact disulfide bond. The concentrations of the rabbit PrP and human PrP were determined by their absorbance at 280 nm using the molar extinction coefficient values of 57,995 and 57,995 M^−1^ cm^−1^, respectively, deduced from the composition of the proteins online.

Human SOD1 mutant A4V was generated from wild-type human SOD1 which cloned in pET3d vector (kindly provided by Dr. Thomas O'Halloran) using primers CTTCAGCACGCACACGACCTTCGTGGCCATGG/CCATGGCCACGAAGGTCG TGTGCGTGCTGAAG. Such a pathological mutant was expressed in *Escherichia coli* and purified to homogeneity by Q-Sepharose chromatography as described [30]. Purified human SOD1 was analyzed by SDS-PAGE with one band. The demetallated (apo) SOD1 was prepared according to previously published protocols [31]. The concentration of human SOD1 was determined according to its absorbance at 280 nm using the molar extinction coefficient value of 10,800 M^−1^ cm^−1^/dimer [31]. Hen egg white lysozyme was obtained from Sigma-Aldrich and was used without further purification. The *A* ^1%^_1 cm_ value of 26.5 at 280 nm [32] was used for protein concentration measurements.

### Phosphorylation of Tau_244–441_

Recombinant Tau_244–441_ (0.5 mg/ml) was phosphorylated by GSK-3β (20 µg/ml) in the phosphorylation solution containing 2 mM ATP, 8 mM MgCl_2_, 5 mM EGTA, 1 mM phenylmethylsulfonyl fluoride, 2 mM DTT, and 60 mM HEPES (pH 7.4) at 37°C for 20 h and terminated by heating the reaction solutions at 95°C for 5 min. The cooled phosphorylated Tau_244–441_ was centrifuged at 10,000 *g* for 10 min to remove protein aggregates and the protein was concentrated and stored at −20°C [11].

### Thioflavin T binding assays

A 2.5 mM ThT stock solution was freshly prepared in 10 mM Tris-HCl buffer (pH 7.5) for human Tau and prepared in phosphate-buffered saline solution (PBS, 140 mM NaCl, 2.7 mM KCl, 10 mM Na_2_HPO_4_, 1.8 mM KH_2_PO_4_, adjusted to pH 7.0) for prion proteins and human SOD1, and passed through a 0.22-µm pore size filter before use to remove insoluble particles. The method for fibrillization of Tau fragments was similar to the method described by the Mandelkow lab [33] with minor changes [11]. 12 µM Tau_244–372_ and GSK-3β phosphorylated Tau_244–441_ were incubated in 10 mM Tris-HCl buffer (pH 7.5) containing 1 mM DTT and 20 µM ThT with or without a crowding agent at 37°C for up to 2 h in the presence of heparin. The method for fibrillization of SOD1 was similar to the method described by the Valentine lab [30] with minor changes. 50 µM apo-SOD1 mutant was incubated at in 37°C in 10 mM NaH_2_PO_4_-Na_2_HPO_4_ buffer (pH 7.4) containing 1 mM DTT in the absence and presence of crowding agents with continuous shaking at 220 rpm, and samples (50 µl) were diluted into NaH_2_PO_4_-Na_2_HPO_4_ buffer containing 62.5 µM ThT, giving a final volume of 500 µl. The fluorescence of ThT was excited at 440 nm with a slit-width of 7.5 nm and the emission was measured at 480 nm with a slit-width of 7.5 nm on an LS-55 luminescence spectrometer (PerkinElmer Life Sciences, Shelton, CT).

The methods for fibrillization of prion proteins were similar to the methods described by the Baskakov lab [29], [34] with minor changes. A stock solution of the human/rabbit PrPs in 6 M GdnHCl was diluted to a final concentration of 10 µM and incubated at 37°C in PBS buffer (pH 7.0) containing 2 M GdnHCl for prion proteins and in PBS buffer containing 1 M GdnHCl and 3 M urea for the rabbit PrP in the absence and presence of crowding agents with continuous shaking at 220 rpm, and samples (50 µl) were diluted into PBS buffer containing 12.5 µM ThT, giving a final volume of 2.5 ml. The fluorescence of ThT was excited at 450 nm with a slit-width of 5/7.5 nm and the emission was measured at 480 nm with a slit-width of 5/7.5 nm for human/rabbit PrPs on an LS-55 luminescence spectrometer.

The method for aggregation of hen lysozyme was similar to the method described by the Dobson lab [35] with minor changes. Hen lysozyme was denatured in HCl solution (pH 2.0) containing 100 mM NaCl and 0.2% NaN_3_ at 37°C for 3–5 days. The lysozyme solution was then mixed with stock solutions of crowding agents, to yield a solution of 350 µM lysozyme in HCl (pH 2.0) containing a chosen concentration of a crowding agent, followed by incubated at 37°C for 14 days with continuous shaking at 220 rpm. Samples (10 µl) were diluted into 10 mM NaH_2_PO_4_-Na_2_HPO_4_ buffer (pH 7.0) containing 100 mM NaCl and 65 µM ThT, giving a final volume of 3 ml. The fluorescence of ThT was excited at 440 nm with a slit-width of 10 nm and the emission was measured at 482 nm with a slit-width of 5 nm on an LS-55 luminescence spectrometer.

Control experiments were performed to ensure that the crowding agents had no influence on the above ThT binding assays.

### Turbidity assays

12 µM Tau fragments (Tau_244–372_ and GSK-3β phosphorylated Tau_244–441_) were incubated without agitation in 10 mM Tris-HCl buffer (pH 7.5) containing 3 µM heparin and 1 mM DTT at 37°C and the solutions were placed into 1-cm path length acryl cuvettes followed by monitoring the turbidity at 400 nm using a UV-2550 Probe spectrophotometer (Shimadzu, Kyoto, Japan). The preparation of the samples before the first measurement took 1 min.

Aggregation of 350 µM hen egg white lysozyme was carried out as stated above, during the incubation time, 500 µl samples were taken out and placed into 1-cm path length acryl cuvettes followed by monitoring the turbidity at 400 nm using a UV-2550 Probe spectrophotometer.

All kinetic experiments were repeated three times. The experiments were pretty reproducible. Every time crowding agents enhanced fibril formation of the three amyloidogenic proteins (human Tau fragments, the human PrP, and human SOD1) and inhibited aggregation of the rabbit PrP and hen egg white lysozyme, although the ThT fluorescence intensities (or the turbidity at 400 nm) were slightly different in different batches.

### Kinetic model

Kinetic parameters were determined by fitting ThT fluorescence intensity *versus* time to the empirical Hill equation [4], [11]:
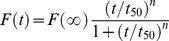
(1)where *F*(∞) is the fluorescence intensity in the long time limit, *t*_50_ is the elapsed time at which *F* is equal to one-half of *F*(∞), and *n* is a cooperativity parameter.

### Sarkosyl-soluble SDS-PAGE

Amyloid formation of 20 µM human/rabbit PrPs and pathological human PrP mutants E196K and D178N was carried out as stated above, during the incubation time, 200 µl samples were taken out and dialyzed against 20 mM sodium acetate buffer (pH 5.0) to remove GdnHCl. Then 25 µl samples were taken out and added with 2.5 µl of 100 mM Tris-HCl (pH 7.0) and 2.5 µl of 20% Sarkosyl. The mixture was left at room temperature for 30 min and then centrifugated at 17,000 *g* for 30 min with an Eppendorf 5810R centrifuge (Eppendorf AG, Hamburg, Germany). The supernatant was taken out and mixed with 2× loading buffer and separated by 13.5% SDS-PAGE. Gel was stained by Coomassie Blue R250.

### Sarkosyl-insoluble SDS-PAGE

Fibrillization of 50 µM apo-SOD1 mutant A4V was carried out as stated above, during the incubation time, 50 µl samples were taken out and added into 50 µl of 10 mM NaH_2_PO_4_-Na_2_HPO_4_ buffer (pH 7.4) containing 10% Sarkosyl. Aggregation of 350 µM hen egg white lysozyme was carried out as stated above, during the incubation time, 50 µl samples were taken out and added into 70 µl of 10 mM NaH_2_PO_4_-Na_2_HPO_4_ buffer (pH 7.0) containing 10% Sarkosyl. The mixtures were left at room temperature for 30 min and then centrifugated on a CS150GXL micro ultracentrifuge (Hitachi, Tokyo, Japan) at 150,000 *g* for 30 min. The supernatant (Sarkosyl-soluble SOD1/lysozyme) was removed, and the pellet (Sarkosyl-insoluble SOD1/lysozyme) was re-suspended in 2× loading buffer and subjected to 13.5% SDS-PAGE. After the electrophoresis the gels were stained with Coomassie Blue R250.

### Transmission electron microscopy

The formation of fibrils by human Tau fragments, by the human/rabbit PrPs, by human SOD1, and by hen egg white lysozyme was confirmed by electron microscopy of negatively stained samples. The incubation time was chosen within a time range of the plateau of each kinetic curve of ThT fluorescence. Sample aliquots of 10 µl were placed on carbon-coated copper grids, and left at room temperature for 1–2 min, rinsed with H_2_O twice, and then stained with 2% (w/v) uranyl acetate for another 1–2 min. The stained samples were examined using an H-8100 (or an H-7000 FA) transmission electron microscope (Hitachi, Tokyo, Japan) operating at 100 kV or an FEI Tecnai G2 20 transmission electron microscope (Hillsboro, OR) operating at 200 kV.

## Results

### Macromolecular crowding enhances fibril formation of amyloidogenic proteins

**Figure 1:**
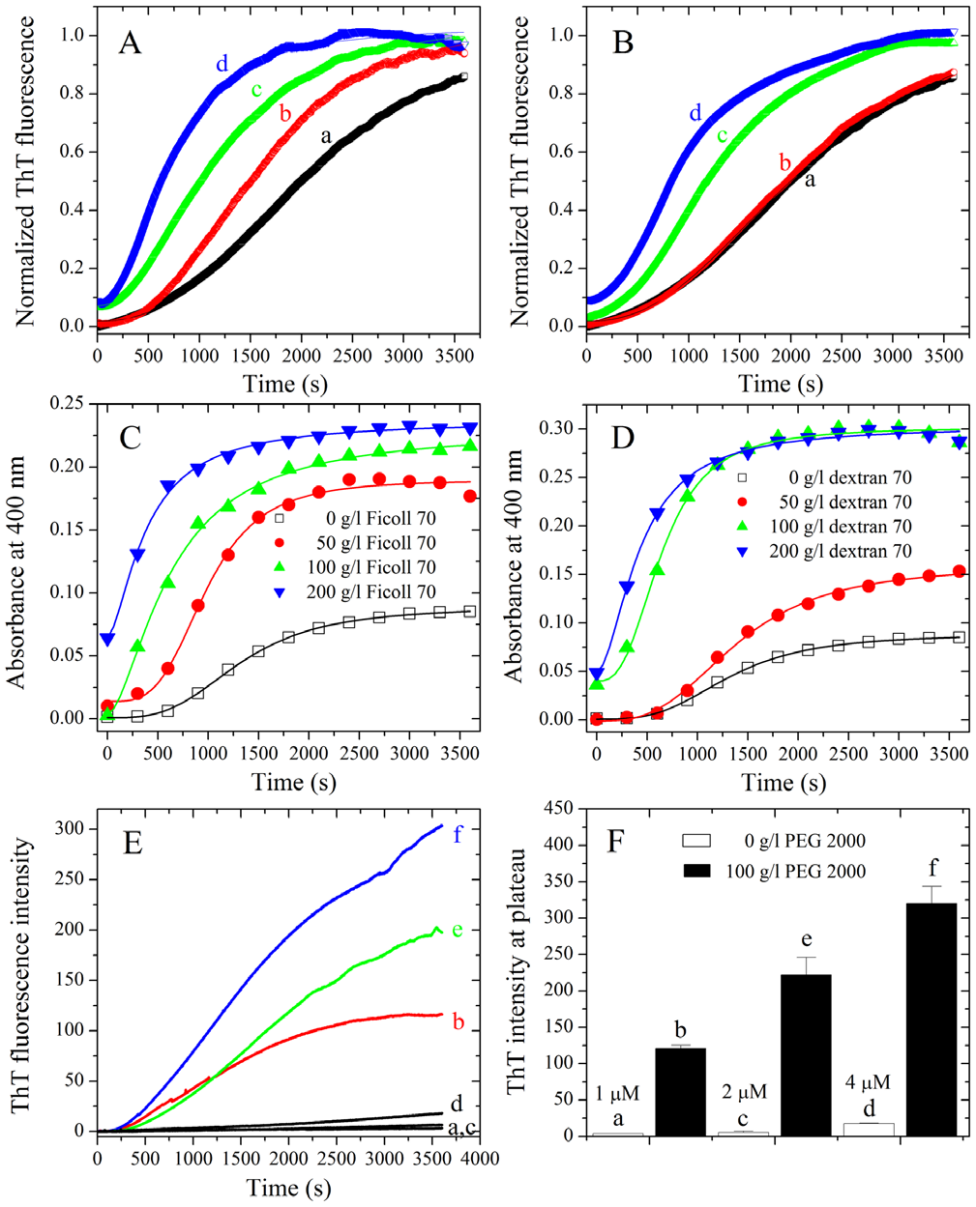
Macromolecular crowding enhances Tau_244–372_ fibrillization. Filament formation of human Tau fragment Tau_244–372_ in the absence and in the presence of Ficoll 70 (A) and dextran 70 (B) respectively, monitored by ThT fluorescence. The crowding agent concentrations were 0 (a), 50 g/l (b), 100 g/l (c), and 200 g/l (d), respectively. Filament formation of Tau_244–372_ in the absence and in the presence of Ficoll 70 (C) and dextran 70 (D) respectively, monitored by turbidity. The crowding agent concentrations were 0 (open square), 50 g/l (solid circle), 100 g/l (solid triangle), and 200 g/l (inverted solid triangle), respectively. The empirical Hill equation was fitted to the data and the solid lines represented the best fit. The final concentration of human Tau fragment was 12 µM. E and F: filament formation of Tau_244–372_ at different concentrations in absence (a, c, and d) and in the presence of 100 g/l PEG 2000 (b, e, and f), represented by ThT fluorescence intensity at plateau. The data with error bars are expressed as the mean ± S.D. (*n* = 3). The assays were carried out at 37°C.

Ficoll 70, Ficoll 400, and dextran 70 are widely accepted as perfect models for the principal crowding components in living cells where the folding and misfolding of proteins take place, because their interactions with proteins can be described using pure excluded-volume models [7], [13]. By contrast, PEG is another kind of crowding agent, whose interactions with proteins cannot be described quantitatively in terms of excluded volume alone [7]. In this study, the effects of three macromolecular crowding agents, Ficoll 70, dextran 70, and PEG 2000, on human Tau filament formation were examined by ThT binding and turbidity assays (Fig. 1), as a function of crowder concentration. Three human Tau fragments, non-phosphorylated Tau_244–372_ and Tau_244–441_, and Tau_244–441_ phosphorylated by GSK-3β, were employed. Effects of added crowding agents on the rate of fibril formation of Tau_244–372_ were monitored *via* measurement of the time-dependent ThT fluorescence (Fig. 1A and 1B) and turbidity (Fig. 1C and 1D). Both measurements indicated that the addition of crowding agents dramatically accelerated fibrillization of human Tau fragments Tau_244–372_.

For most protein aggregation systems, increasing concentration of the protein results in increased rates of aggregation. Typical data for protein concentration-dependent ThT fluorescence of Tau_244–372_ are shown in Figure 1E and 1F. As shown in Figure 1E and 1F, there was no observed increase of ThT fluorescence up to 1 h when Tau_244–372_ is 1 µM, and by increasing the concentration of Tau_244–372_ up to 4 µM, both the rates of filament formation and the maximum intensity of the ThT fluorescence increased. But the presence of 100 g/l PEG 2000 promoted the process even at 1 µM, and enhanced both the overall rates of the reaction and the maximum intensity of ThT fluorescence.

**Figure 2:**
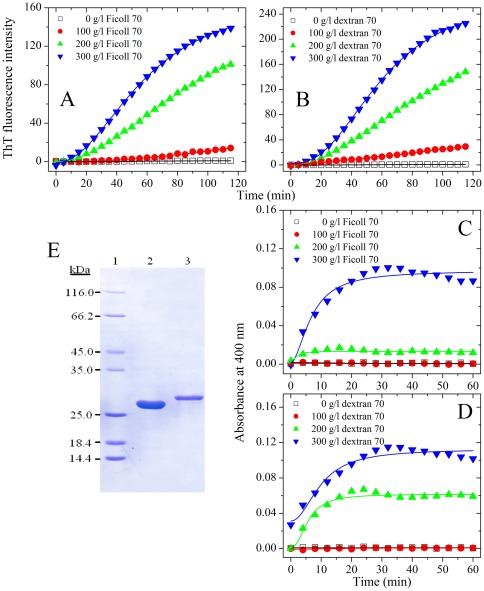
Macromolecular crowding enhances GSK-3β phosphorylated Tau_244–441_ fibrillization. Filament formation of GSK-3β phosphorylated Tau_244–441_ in the absence and in the presence of Ficoll 70 (A and C) and dextran 70 (B and D), respectively, monitored by ThT fluorescence (A and B) and turbidity (C and D). The crowding agent concentrations were 0 (open square), 100 g/l (solid circle), 200 g/l (solid triangle), and 300 g/l (inverted solid triangle), respectively. The empirical Hill equation was fitted to the data and the solid lines represented the best fit. The final concentration of human Tau fragment was 12 µM. The assays were carried out at 37°C. (E) SDS-PAGE profiles of non-phosphorylated and GSK-3β phosphorylated Tau_244–441_ fragments. Lane 1, molecular weight SDS calibration kit protein standards. Lane 2 represents non-phosphorylated Tau_244–441_ and lane 3 represents GSK-3β phosphorylated Tau_244–441_. Proteins in the gel were visualized by a Coomassie Brilliant Blue R staining.

SDS-PAGE profiles of Tau_244–441_ and Tau_244–441_ phosphorylated by GSK-3β are shown in Figure 2E. Nonphosphorylated Tau_244–441_ (lane 2) migrated as a single band, and phosphorylated Tau_244–441_ (lane 3) migrated slower. As revealed by assays based on ThT binding (Figure 2A and 2B) and turbidity (Figure 2C and 2D), human Tau fragment Tau_244–441_, when phosphorylated by GSK-3β, did not form filaments in the absence of a crowding agent but did form fibrils in the presence of a crowding agent (Ficoll 70 or dextran 70). The above results suggest a compensation mechanism of macromolecular crowding to the lost capability of fibril formation caused by the phosphorylation of Tau and phosphorylated Tau associated with Alzheimer disease is more likely to form amyloid fibrils under crowded conditions than in dilute solutions. An alternative explanation for the above results is that macromolecular crowding is accelerating a process that by phosphorylation might have been retarded.

**Figure 3:**
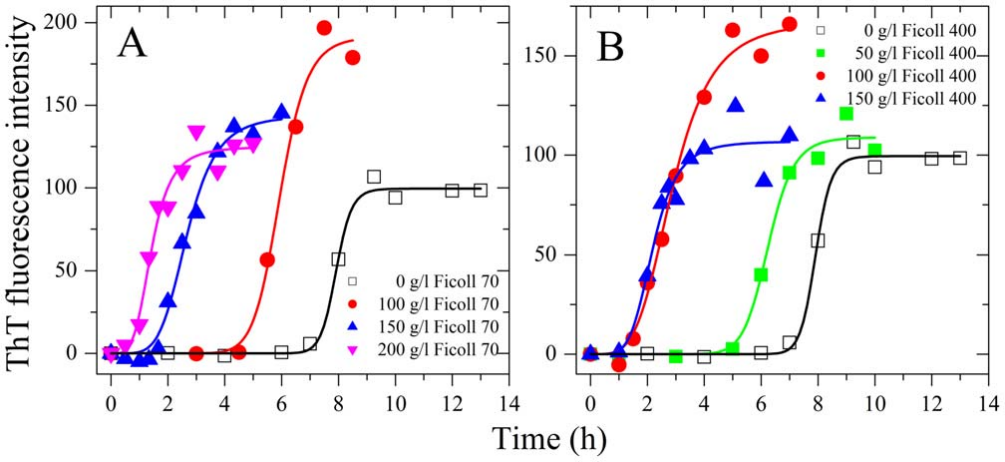
Macromolecular crowding enhances amyloid fibril formation of human prion protein. Fibril formation of human prion protein in the absence and in the presence of Ficoll 70 (A) and Ficoll 400 (B), respectively, monitored by ThT fluorescence. The empirical Hill equation was fitted to the data and the solid lines represented the best fit. The final concentration of human PrP was 10 µM. The crowding agent concentrations were 0 (open square), 50 g/l (solid square), 100 g/l (solid circle), 150 g/l (solid triangle), and 200 g/l (inverted solid triangle), respectively. The human PrP was denatured in PBS buffer (pH 7.0) containing 2 M GdnHCl. The assays were carried out at 37°C.

**Figure 4:**
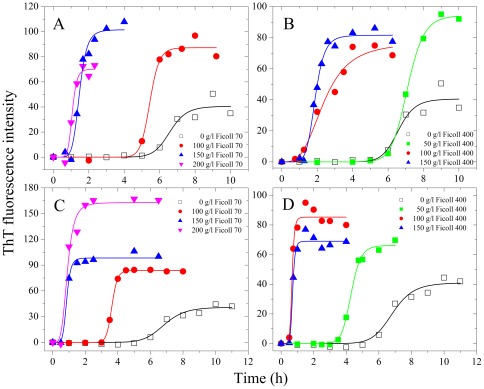
Macromolecular crowding enhances amyloid fibril formation of pathological human prion protein mutants. Fibril formation of pathogenic mutant E196K in the absence and in the presence of Ficoll 70 (A) and Ficoll 400 (B), respectively, and another pathogenic mutant D178N in the absence and in the presence of Ficoll 70 (C) and Ficoll 400 (D), respectively, monitored by ThT fluorescence. The empirical Hill equation was fitted to the data and the solid lines represented the best fit. The final concentrations of human PrP mutants were 10 µM. The crowding agent concentrations were 0 (open square), 50 g/l (solid square), 100 g/l (solid circle), 150 g/l (solid triangle), and 200 g/l (inverted solid triangle), respectively. The assays were carried out at 37°C.

Human familial prion diseases are associated with about 40 point mutations of the gene coding for the prion protein [16], [22], [36]. We then investigated the effects of macromolecular crowding on fibril formation of the human PrP and its pathogenic mutants E196K and D178N. As shown in Figure 3A and Figure 4A and 4C, the presence of Ficoll 70 at concentrations of 100–200 g/l in the reaction systems significantly accelerated amyloid formation of the human PrP and its pathogenic mutants E196K and D178N on the investigated time scale. Similarly, the presence of Ficoll 400 at 50–150 g/l in the reaction systems also significantly accelerated fibril formation of the human PrP and its pathogenic mutants on the investigated time scale (Fig. 3B and Fig. 4B and 4D). Furthermore, the enhancing effect of Ficoll 400 on fibril formation of the human PrP and its pathogenic mutants was stronger than that of Ficoll 70 (Figs. 3 and 4). Clearly, the presence of a strong crowding agent dramatically promoted amyloid fibril formation of human prion protein and its two pathogenic mutants E196K and D178N (Figs. 3 and 4).

**Figure 5:**
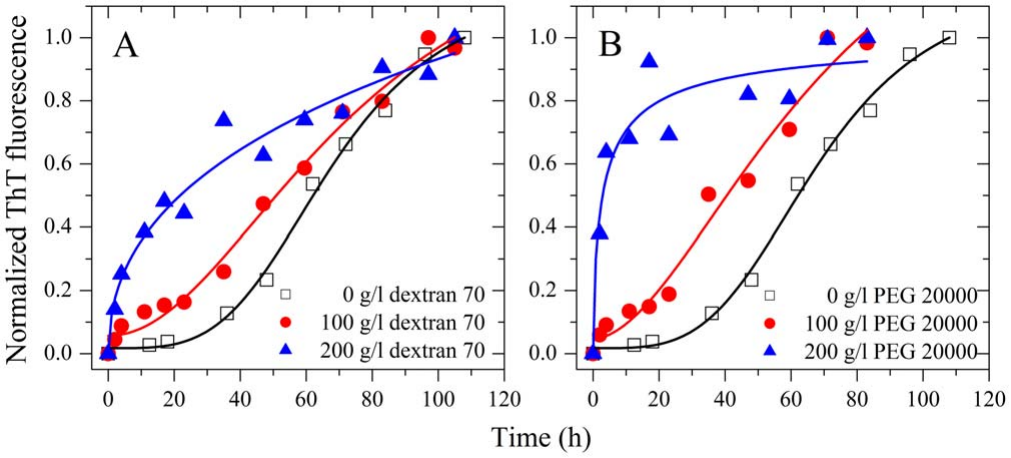
Macromolecular crowding enhances fibril formation of pathological human SOD1 mutant. Fibril formation of pathogenic mutant A4V in the absence and in the presence of dextran 70 (A) and PEG 20000 (B), respectively, monitored by ThT fluorescence. The empirical Hill equation was fitted to the data and the solid lines represented the best fit. The final concentration of human SOD1 mutant was 50 µM. The crowding agent concentrations were 0 (open square), 100 g/l (solid circle), and 200 g/l (solid triangle), respectively. The assays were carried out at 37°C.

Pathological human SOD1 mutant A4V is the most common familial ALS mutation in North America and has a particularly short disease duration [37]. We finally investigated the effects of macromolecular crowding on fibril formation of such a pathogenic mutant. As shown in Figure 5A and 5B, the presence of dextran 70 or PEG 20000 at concentrations of 100–200 g/l in the reaction systems significantly accelerated fibril formation of A4V on the investigated time scale. Furthermore, the enhancing effect of PEG 20000 on fibril formation of the SOD1 mutant was stronger than that of dextran 70 (Fig. 5). Clearly, an enhancing effect of macromolecular crowding on fibril formation is also observed for a pathological human SOD1 mutant A4V (Fig. 5).

### Macromolecular crowding inhibits aggregation of some non-amyloidogenic proteins

**Figure 6:**
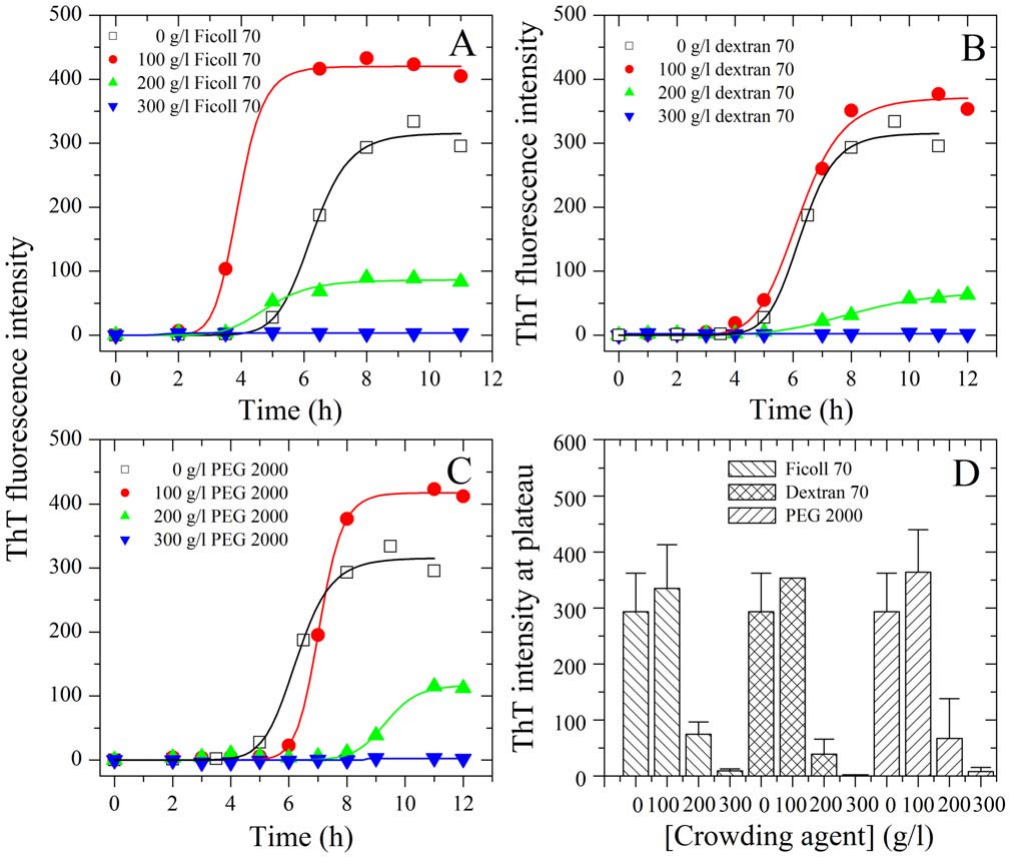
Macromolecular crowding inhibits aggregation formation of rabbit prion protein. Aggregation of rabbit prion protein in the absence and in the presence of Ficoll 70 (A), dextran 70 (B), and PEG 2000 (C), respectively, monitored by ThT fluorescence. The empirical Hill equation was fitted to the data and the solid lines represented the best fit. The final concentration of rabbit PrP was 10 µM. The crowding agent concentrations were 0 (open square), 100 g/l (solid circle), 200 g/l (solid triangle), and 300 g/l (inverted solid triangle), respectively. (D) Effects of macromolecular crowding on ThT fluorescence intensity of rabbit PrP fibrils at plateau in absence and in the presence of Ficoll 70, dextran 70 or PEG 2000. The crowding agent concentrations were 0 (the first column), 100 g/l (the second column), 200 g/l (the third column), and 300 g/l (the fourth column), respectively. The data with error bars are expressed as the mean ± S.D. (*n* = 3). The rabbit PrP was denatured in PBS buffer (pH 7.0) containing 1 M GdnHCl and 3 M urea. The assays were carried out at 37°C.

For the rabbit PrP, time dependence of ThT fluorescence as a function of crowder concentration is shown in Figures 6 and S1. Effects of crowding agents on amyloid fibril formation of the rabbit PrP denatured in PBS buffer containing 1 M GdnHCl and 3 M urea (or denatured in PBS buffer containing 2 M GdnHCl, the same conditions as those of the human PrP) depended on the concentrations of the crowding agents. As shown in Figures 6 and S1A–C, the rabbit PrP did not form amyloid fibrils when a crowding agent (Ficoll 70, dextran 70 or PEG 2000) at 300 g/l is used but did form fibrils in the absence of a crowding agent. Furthermore, aggregation of the rabbit PrP was remarkably inhibited by Ficoll 70, dextran 70 and PEG 2000 at 200 g/l on the investigated time scale, accompanied by a remarkable decline of the maximum ThT intensity (Fig. 6D), while the presence of any of the three crowding agents at 100 g/l promoted aggregation of the rabbit PrP denatured by 1 M GdnHCl and 3 M urea to some extent (Fig. 6) or inhibited aggregation of the rabbit PrP denatured by 2 M GdnHCl (Fig. S1). In addition, the inhibitory effect of dextran 70 on aggregation of the rabbit PrP was stronger than that of Ficoll 70 (Fig. 6A and 6B).

**Figure 7:**
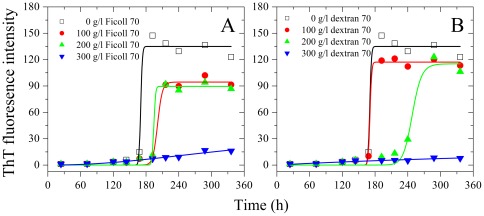
Macromolecular crowding inhibits aggregation formation of hen egg white lysozyme. Aggregation of hen egg white lysozyme in the absence and in the presence of Ficoll 70 (A) and dextran 70 (B), respectively, monitored by ThT fluorescence. The empirical Hill equation was fitted to the data and the solid lines represented the best fit. The final concentration of hen lysozyme was 350 µM. The crowding agent concentrations were 0 (open square), 100 g/l (solid circle), 200 g/l (solid triangle), and 300 g/l (inverted solid triangle), respectively. The assays were carried out at 37°C.

We then investigated the effects of macromolecular crowding on amyloid formation of hen egg white lysozyme, another non-amyloidogenic protein. For hen lysozyme, time dependence of ThT fluorescence as a function of crowder concentration is shown in Figure 7. As shown in Figure 7A and 7B, hen lysozyme almost did not form amyloid fibrils when a crowding agent (Ficoll 70 or dextran 70) at 300 g/l is used but did form fibrils in the absence of a crowding agent. Furthermore, aggregation of hen lysozyme was markedly inhibited by Ficoll 70 and dextran 70 at 200 g/l on the investigated time scale, accompanied by a decline of the maximum ThT intensity, while the presence of 100 g/l dextran 70 almost did not inhibit aggregation of hen lysozyme (Fig. 7). Time-dependent turbidity of hen lysozyme as a function of crowder concentration is shown in Figure S2. As shown in Figure S2, hen lysozyme almost did not form aggregates when 300 g/l Ficoll 70 is used but did form aggregates in the absence of a crowding agent, further supporting the conclusion reached by ThT binding assays that macromolecular crowding remarkably inhibits aggregation of hen lysozyme.

### The amount of protein fibrils/monomers present in the solution measured by centrifugation assays

**Figure 8:**
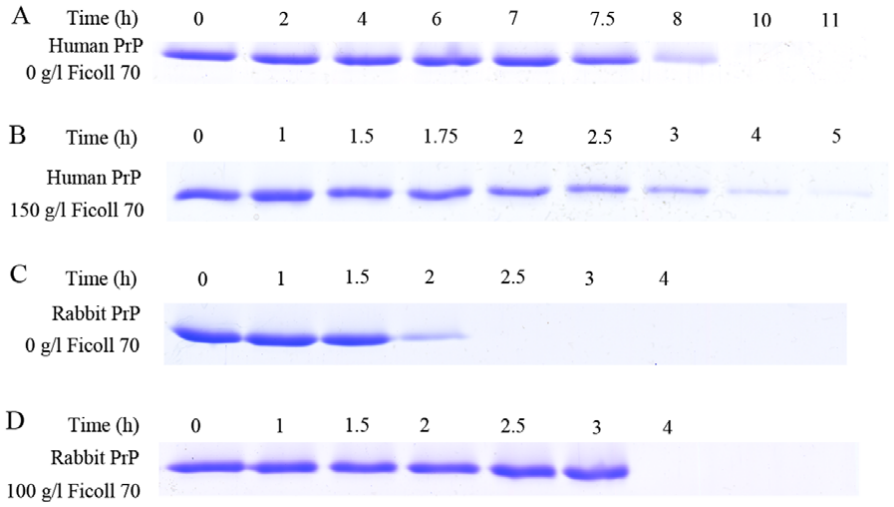
Time-dependent SDS-PAGE analysis of Sarkosyl-soluble human prion protein (A and B) and rabbit prion protein (C and D) incubated in 0 g/l (A and C), 150 g/l (B), and 100 g/l (D) Ficoll 70. Samples were taken and dialyzed against 20 mM sodium acetate buffer, and incubated with 100 mM Tris-HCl buffer containing 2% Sarkosyl for 30 min. Then the samples were centrifugated at 17,000 *g* for 30 min and the supernatants were mixed with 2× loading buffer and separated by 13.5% SDS-PAGE. Gel was stained by Coomassie Blue R250. The human/rabbit PrPs were denatured in PBS buffer (pH 7.0) containing 2 M GdnHCl.

**Figure 9:**
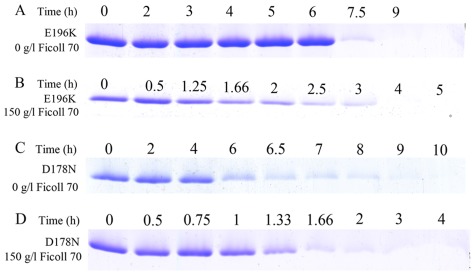
Time-dependent SDS-PAGE analysis of Sarkosyl-soluble pathological human prion protein mutants E196K (A and B) and D178N (C and D) incubated in 0 g/l (A and C) and 150 g/l (B and D) Ficoll 70. Samples were taken and dialyzed against 20 mM sodium acetate buffer, and incubated with 100 mM Tris-HCl buffer containing 2% Sarkosyl for 30 min. Then the samples were centrifugated at 17,000 *g* for 30 min and the supernatants were mixed with 2× loading buffer and separated by 13.5% SDS-PAGE. Gel was stained by Coomassie Blue R250.

ThT fluorescence is not perfectly specific for amyloid fibrils and, depending on the particular protein and experimental conditions, assays may render both false positive (spectroscopic change upon binding to non-fibrillar material) and false negative results (its fluorescence not being affected by some amyloid fibrils). Considering this, we investigated the correlation between the spectroscopic signal monitored and the amount of protein fibrils/monomers present in the solution measured by centrifugation assays. In order to semi-quantify the decrease/increase of monomeric proteins in the presence of crowding agents, we carried out Sarkosyl-soluble SDS-PAGE experiments after centrifugation assays. As shown in Figure 8A and 8B, a clear band corresponding to Sarkosyl-soluble human PrP monomers was observed when the human PrP was incubated in the absence of a crowding agent for 8 h, while the human PrP monomer band was observed when the human PrP was incubated with 150 g/l Ficoll 70 for a remarkably shorter time (2–4 h). As shown in Figure 9A–D, a clear band corresponding to Sarkosyl-soluble human PrP monomers was observed when pathological human PrP mutants E196K and D178N were incubated in the absence of a crowding agent for around 7 h, while the human PrP monomer band was observed when E196K and D178N were incubated with 150 g/l Ficoll 70 for a much shorter time (2–3 h for E196K and 1–2 h for D178N). As shown in Figure 8C and 8D, a clear band corresponding to Sarkosyl-soluble rabbit PrP monomers was observed when the rabbit protein was incubated with 100 g/l Ficoll 70 for 3 h, while the rabbit PrP monomer band was observed when the rabbit protein was incubated in the absence of a crowding agent for a shorter time (2 h). The above results indicate that while crowding agents dramatically promote fibril formation of human PrP and its two pathogenic mutants E196K and D178N, they inhibit aggregation of the rabbit PrP by stabilizing its native state.

**Figure 10:**
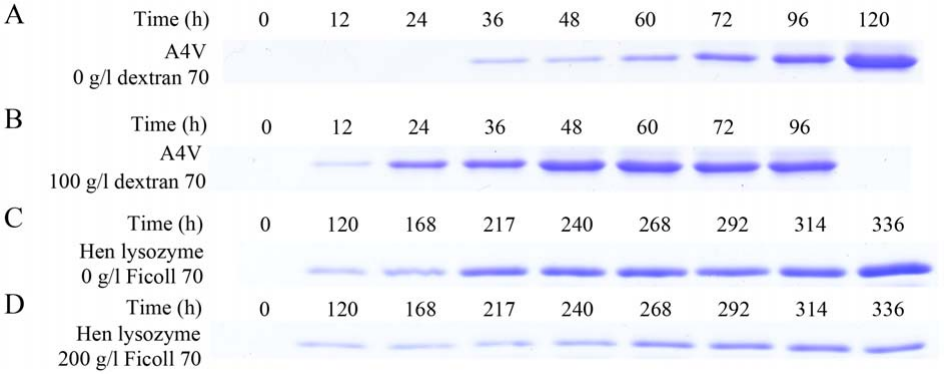
Time-dependent SDS-PAGE analysis of Sarkosyl-insoluble pathological human SOD1 mutant A4V (A and B) and hen egg white lysozyme (C and D) incubated in 0 g/l (A and C) and 100 g/l dextran 70 (B) or 200 g/l Ficoll 70 (D). Samples were taken and incubated with 10 mM NaH_2_PO_4_-Na_2_HPO_4_ buffer containing 10% Sarkosyl for 30 min followed by centrifuging at 150,000 *g* for 30 min. Pellets were re-suspended with 2× loading buffer and separated by 13.5% SDS-PAGE. Gel was stained by Coomassie Blue R250.

In order to semi-quantify the increase/decrease of protein fibrils in the presence of crowding agents, we carried out Sarkosyl-insoluble SDS-PAGE experiments after centrifugation assays. As shown in Figure 10A and 10B, a clear band corresponding to Sarkosyl-insoluble SOD1 fibrils was observed when pathological human SOD1 mutant A4V was incubated in the absence of a crowding agent for 36 h, while the Sarkosyl-insoluble SOD1 band was observed when A4V was incubated with 100 g/l dextran 70 for a much shorter time (12 h). Furthermore, when A4V was incubated for 48 h, the intensity of the Sarkosyl-insoluble SOD1 band in the presence of 100 g/l dextran 70 was remarkably higher than that in the absence of a crowding agent. As shown in Figure 10C and 10D, a clear band corresponding to Sarkosyl-insoluble lysozyme fibrils was observed when hen egg white lysozyme was incubated without or with 200 g/l Ficoll 70 for 120 h. When hen lysozyme was incubated for 217/240/268/292/314/336 h, the intensity of the Sarkosyl-insoluble lysozyme band in the presence of 200 g/l Ficoll 70 was remarkably lower than that in the absence of a crowding agent (Fig. 10C and 10D). The above results indicate that while crowding agents significantly promote fibril formation of pathological human SOD1 mutant A4V, they inhibit aggregation of hen lysozyme by stabilizing its native conformation.

### Characterization of the morphology of protein aggregates formed in the presence of crowding agents

**Figure 11:**
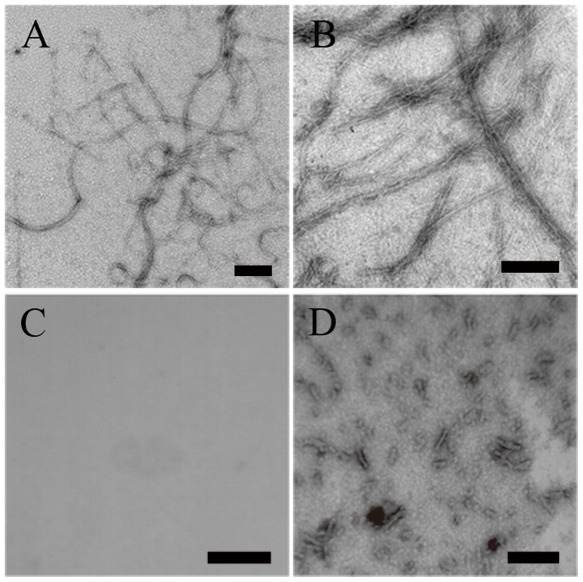
Transmission electron micrographs of human Tau fragment samples at physiological pH after incubation under different conditions. Tau_244–372_ (A and B) and GSK-3β phosphorylated Tau_244–441_ (C and D) samples were incubated for 1 h (A and B) or 2 h (C and D) in the absence of a crowding agent (A and C) and in the presence of 150 g/l Ficoll 70 (B) or 300 g/l Ficoll 70 (D), respectively. A 2% (w/v) uranyl acetate solution was used to negatively stain the fibrils. The scale bars represent 200 nm.

**Figure 12:**
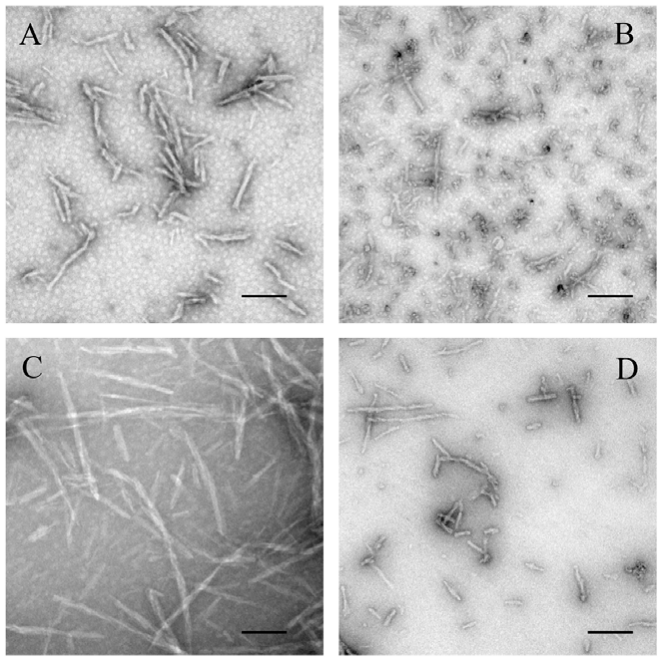
Transmission electron micrographs of human/rabbit PrP samples at physiological pH after incubation under different conditions. Human (A and B) and rabbit (C and D) PrP samples were incubated for 9 h (A) or 3 h (B, C, and D) in the absence of a crowding agent (A and C) and in the presence of 150 g/l Ficoll 70 (B) or 200 g/l Ficoll 70 (D), respectively. A 2% (w/v) uranyl acetate solution was used to negatively stain the fibrils. The scale bars represent 200 nm. The human/rabbit PrPs were denatured in PBS buffer (pH 7.0) containing 2 M GdnHCl.

**Figure 13:**
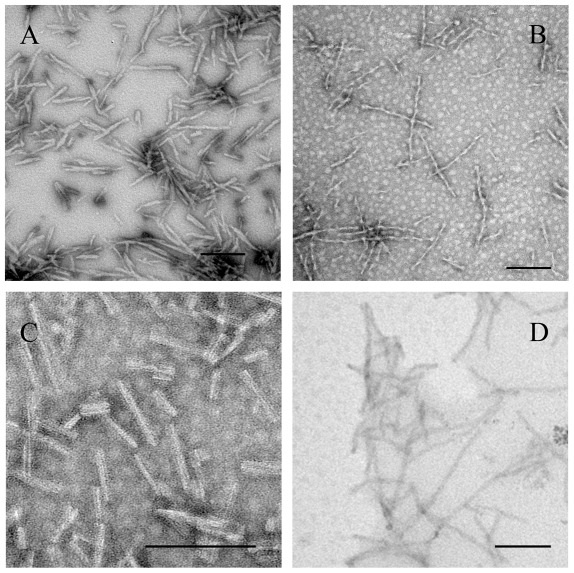
Transmission electron micrographs of pathological human PrP mutant samples at physiological pH after incubation under different conditions. Pathogenic mutant E196K (A and B) and D178N (C and D) samples were incubated for 8 h (A and C) or 2 h (B and D) in the absence of a crowding agent (A and C) and in the presence of 150 g/l Ficoll 70 (B and D), respectively. A 2% (w/v) uranyl acetate solution was used to negatively stain the fibrils. The scale bars represent 200 nm.

**Figure 14:**
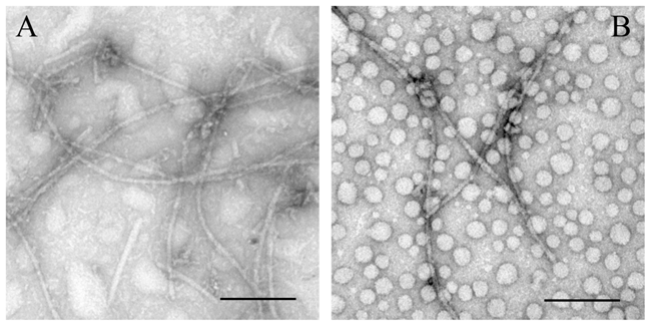
Transmission electron micrographs of pathological human SOD1 mutant samples at physiological pH after incubation under different conditions. Pathogenic mutant A4V samples were incubated for 108 h in the absence of a crowding agent (A) and in the presence of 100 g/l dextran 70 (B), respectively. A 2% (w/v) uranyl acetate solution was used to negatively stain the fibrils. The scale bars represent 200 nm.

**Figure 15:**
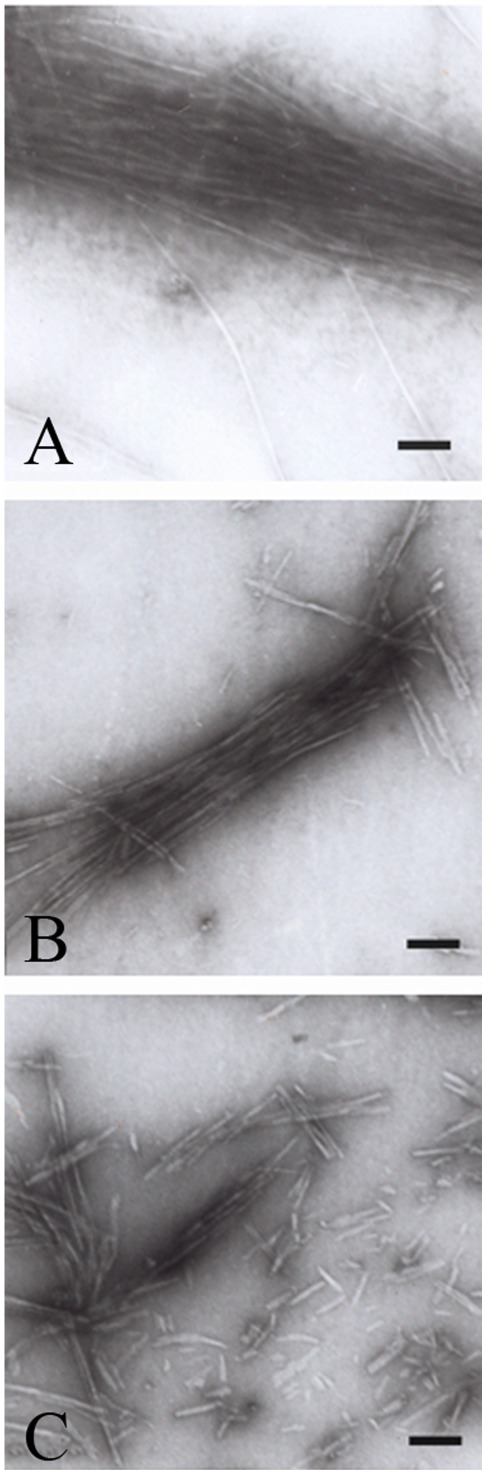
Transmission electron micrographs of hen egg white lysozyme samples at pH 2.0 after incubation under different conditions. Hen lysozyme samples were incubated for 14 days in the absence of a crowding agent (A) and in the presence of 100 g/l Ficoll 70 (B), or 200 g/l Ficoll 70 (C), respectively. A 2% (w/v) uranyl acetate solution was used to negatively stain the fibrils. The scale bars represent 200 nm.

TEM was employed to characterize the morphology of protein aggregates formed in the absence and in the presence of crowding agents. Figures 11, 12, 13, 14 and 15 show TEM images of Tau fragment samples, human/rabbit PrP samples, pathological human PrP mutant samples, pathological human SOD1 mutant samples, and hen lysozyme samples incubated in the solution of a crowding agent (Ficoll 70 or dextran 70). For non-phosphorylated Tau_244–372_, the addition of 150 g/l Ficoll 70 had no significant effect on the morphology of Tau samples, and long and branched fibrils as well as straight filaments were observed in both samples (Fig. 11A and 11B). In the presence of 300 g/l Ficoll 70, the majority of GSK-3β phosphorylated Tau_244–441_ was observed as short amyloid fibrils (Fig. 11D), but no fibrils were observed for phosphorylated Tau_244–441_ in the absence of a crowder (Fig. 11C), further supporting the conclusion reached by ThT binding and turbidity assays that macromolecular crowding dramatically promotes fibril formation of GSK-3β phosphorylated Tau_244–441_. In absence of a crowding agent, the human PrP formed fibrils with a length of 100–300 nm after incubation for 9 h (Fig. 12A). In the presence of 150 g/l Ficoll 70, however, abundant short amyloid fibrils and spherical or ellipsoidal particles were observed when human PrP samples were incubated for 3 h (Fig. 12B). In absence of a crowding agent, the fibrils formed by the rabbit PrP appear long and twisted structure after incubation for 3 h (Fig. 12C). In the presence of 200 g/l Ficoll 70, however, some short amyloid fibrils and a few fibrils with a length of 100–300 nm were observed when rabbit PrP samples were incubated for 3 h (Fig. 12D). The amount of fibrils formed by the rabbit PrP in the presence of 200 g/l Ficoll 70 (Fig. 12D) appears to be markedly less than that in the absence of a crowding agent (Fig. 12C) on the same time scale, further supporting the conclusion reached by ThT binding assays that macromolecular crowding remarkably inhibits aggregation of the rabbit PrP. The addition of 150 g/l Ficoll 70 had no significant effect on the morphology of pathological human PrP mutant E196K and D178N samples, and many fibrils with a length of 100–300 nm were observed in these samples (Fig. 13A–D). Similarly, the addition of 100 g/l dextran 70 had no significant effect on the morphology of pathological human SOD1 mutant A4V samples, and long and curved fibrils as well as non-fibrillar material were observed in both samples (Fig. 14A and 14B). The addition of 100 g/l Ficoll 70 had no significant effect on the morphology of hen egg white lysozyme samples, and long and bundled fibrils were observed in both samples (Fig. 15A and 15B). In the presence of 200 g/l Ficoll 70, however, hen lysozyme formed abundant fibrils with a length of 100–500 nm as well as some short amyloid fibrils (Fig. 15C). Clearly, fibrils of different proteins formed in the presence of the same crowder (for example, Ficoll 70) were of different morphologies.

## Discussion

Ficoll 70, Ficoll 400, and dextran 70 are widely used to mimic the excluded-volume effects in crowded physiological environments [7], [13]. Compared with dextran 70, Ficoll 70 behaves much more like a rigid sphere with a radius around 55 Å [10], [38]. Ficoll 70 or Ficoll 400 is a highly branched copolymer of two short building blocks, sucrose and epichlorohydrin, making it less flexible and more compact than dextran 70 on a molecular weight basis, and the molecular weight of Ficoll 400 is much larger than that of Ficoll 70. In contrast, dextran 70, a flexible, long-chain poly (D-glucose) with sparse, short branches, is not usually considered a rod-like polymer like double-stranded DNA is and better modeled as a rod-like particle [10], [38]. In the present study, we found that the enhancing effect of Ficoll 400 on fibril formation of the human PrP was stronger than that of Ficoll 70 and the inhibitory effect of dextran 70 on aggregation of the rabbit PrP was stronger than that of Ficoll 70, supporting the conclusion that crowder size and shape are important factors that modulate the net effect of macromolecular crowding on proteins [38].

Our data indicate opposite effects of macromolecular crowding on different proteins, and provide clues to the question of whether the effects of macromolecular crowding on protein misfolding obey a universal rule or must be understood on a case-by-case basis.

The molecular details of protein misfolding are in general not well understood, owing to the complexity and variability of aggregation reactions and technical difficulties in characterizing aggregates, due to their often heterogeneous and fibrillar nature [39]. As mentioned above, amyloid fibrils associated with neurodegenerative diseases can be considered biologically relevant failures of cellular quality control mechanisms. It is known that *in vivo* human Tau protein [17], [20], the human PrP and its pathogenic mutants [16], [21], [22], [36], and human SOD1 pathogenic mutants [18], [23], [24] have the tendency to form fibril deposits in a variety of tissues and thereby cause Alzheimer disease, prion disease, and ALS, respectively, while the rabbit PrP [13], [25], [26] and hen egg white lysozyme [27] do not readily form fibrils and are unlikely to cause neurodegenerative diseases. In the present study, we demonstrated that macromolecular crowding dramatically promoted fibril formation of amyloidogenic proteins, such as GSK-3β phosphorylated human Tau protein, the human PrP and its pathogenic mutants E196K and D178N, and pathological human SOD1 mutant A4V, but remarkably inhibited aggregation of some non-amyloidogenic proteins, such as the rabbit PrP and hen egg white lysozyme. Human Tau protein is a natively unfolded protein [17], [40] so that the most probable path to be followed in the presence of crowders is the formation of compact and stable fibrils as we demonstrate here. However, there are many natively unfolded proteins (for example, histones and transcription factors) that are known to be non-amyloidogenic. Crowding agents have been shown to promote aggregation of some of these proteins (for example, histones) [14] although not of the others [15]. In the case of folded proteins, where the starting materials are denatured proteins, a competition between folding and aggregation is established. The human PrP is a stable folded protein with a long, flexible N-terminal tail [41], and pathological mutants E196K [42], D178N [43], and A4V [44] are all folded proteins with reduced stability, so that macromolecular crowding enhances aggregation of these not-particularly-stable proteins more than folding. However, both the rabbit PrP and hen lysozyme are exceptionally stable folded proteins so that macromolecular crowding stabilizes their native conformations, enhancing folding of these proteins more than aggregation. We thus suggest a contrasting effect of macromolecular crowding on amyloid fibril formation: proteins associated with neurodegenerative diseases are more likely to form amyloid fibrils under crowded conditions than in dilute solutions; by contrast, some of the proteins that are not neurodegenerative disease-associated are unlikely to aggregate and to form amyloid fibrils in crowded physiological environments. Therefore macromolecular crowding could play an important role in the cellular quality control mechanisms.

**Figure 16:**
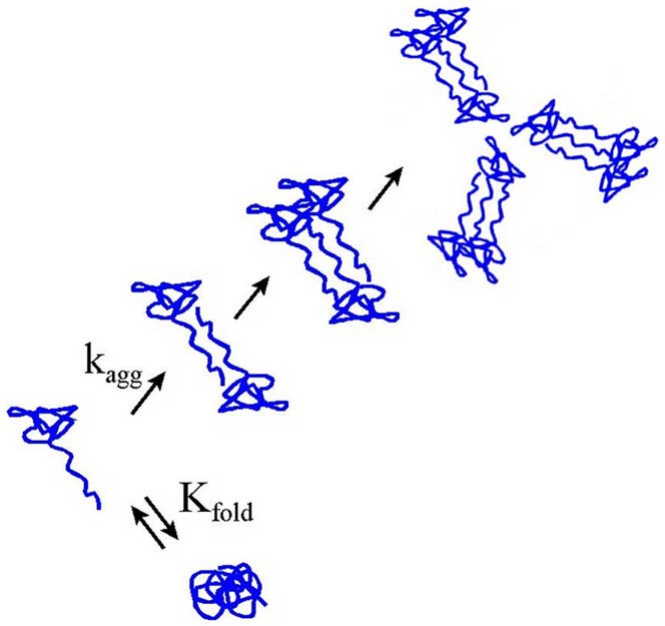
Scheme describing the competition between a conformational transition and an aggregation reaction of a protein in the presence of a crowding agent.

A possible explanation for the contrasting effect of macromolecular crowding on these two sets of proteins (amyloidogenic proteins and non-amyloidogenic proteins) has been proposed. The promotion of human Tau fragments, the human PrP and its pathogenic mutants, and pathological human SOD1 mutant into fibrils by macromolecular crowding is largely caused by the stabilization of intermolecular dimers [7] and the enhancement of the assembly of human Tau, human prion, and human SOD1 molecules by macromolecular crowding. The observations that fibril formation of the rabbit PrP and hen lysozyme are uninhibited remarkably at low crowder concentration and suppressed at higher crowder concentration are interesting and are possibly attributed to the competition between a conformational transition and an aggregation reaction along the lines of the scheme proposed (Fig. 16). Crowder would be expected to increase both *k*_agg_, the rate constant for aggregation, and *K*_fold_, the equilibrium constant for compaction to more folded state that does not aggregate. What might be happening here is that crowder initially increases *k*_agg_, and also increases *K*_fold_, but not enough to increase the equilibrium fraction of the folded state. As the amount of crowder increases, *k*_agg_ continues to increase, but at some point the equilibrium between partially and fully folded monomer shifts toward the fully folded state and then aggregation is disfavored because even though the rate constant for aggregation is higher, the concentration of unfolded monomer, which is the substrate for aggregation is diminished.

Hen egg white lysozyme was used at a concentration of 350 µM, which is much higher than that of other proteins. Such high concentration was used because of the following reasons. Firstly, aggregation of hen lysozyme is concentration-dependent, and increasing concentration of the enzyme could increase the rate of aggregation and facilitate the following measurements, considering it usually takes weeks to form amyloid fibrils from a solution of hen lysozyme (1 mM) at pH 2.0 [35]. Secondly, the concentration of hen lysozyme we used (5 g/l) is one order of magnitude lower than that of a crowding agent (50–300 g/l), which is not enough to create crowding effect on its own.

Misfolding or/and aggregation is an inevitable outcome of a protein's life [1], [20]. For non-amyloidogenic proteins, the resulting molecules are normally cleared by cellular quality control mechanisms [19], [20] in combination with the strong inhibition of fibrillization of such proteins by the crowded physiological environment, thereby not causing any neurodegenerative diseases. For amyloidogenic proteins, however, the combined effects of accelerated production owing to elevated oxidative stress and protein crowding and reduced ability of cells to degrade damaged proteins increase protein aggregation [11], [20]. Incomplete degradation of aggregates can result in production of smaller fragments that can serve as seeds for further aggregation, thereby increasing aggregate burden and causing neurodegenerative diseases [11], [20]. In brief, keeping balance is healthful, but losing balance causes diseases.

The enhancing effect of macromolecular crowding on amyloidogenic protein misfolding is a double-edged sword. On the one hand, a crowded physiological environment could play an exacerbating role in the pathogenesis of neurodegenerative diseases by accelerating amyloidogenic protein misfolding and inducing human prion fibril fragmentation which is considered to be an essential step in prion replication [45]. On the other hand, it has been reported that soluble oligomers and/or protofibrils formed by amyloidogenic proteins are actually the pathogenic species and that fibrils are innocuous (or less toxic/infectious) [46]–[48], and thus a crowded physiological environment could play a neuroprotective role in the onset and progression of neurodegenerative diseases by inducing the most toxic amyloidogenic protein oligomers and/or protofibrils to form innocuous amyloid fibrils.

In conclusion we have shown that: (i) human Tau fragments, when phosphorylated by GSK-3β, do not form filaments in the absence of a crowding agent but do form fibrils in the presence of a crowding agent (Ficoll 70 or dextran 70), and the presence of a strong crowding agent dramatically promotes amyloid fibril formation of the human PrP and its two pathogenic mutants E196K and D178N; (ii) such an enhancing effect of macromolecular crowding on fibril formation is also observed for a pathological human SOD1 mutant A4V; (iii) the rabbit PrP and hen egg white lysozyme do not form amyloid fibrils when a crowding agent (Ficoll 70 or dextran 70) at 300 g/l is used but do form fibrils in the absence of a crowding agent; (iv) aggregation of these two proteins is remarkably inhibited by Ficoll 70 and dextran 70 at 200 g/l on the investigated time scale. Information obtained from the present study can enhance our understanding of the molecular mechanisms of neurodegenerative diseases such as Alzheimer disease, prion disease, and ALS, and should lead to a better understanding of how proteins misfold and how proteins avoid misfolding in crowded physiological environments.

## Supporting Information

Figure S1 **Macromolecular crowding inhibits amyloid fibril formation of rabbit prion protein.** Amyloid formation of rabbit prion protein in the absence and in the presence of Ficoll 70 (A), dextran 70 (B), and PEG 2000 (C), respectively, monitored by ThT fluorescence. The empirical Hill equation was fitted to the data and the solid lines represented the best fit. The final concentration of rabbit PrP was 10 µM. The crowding agent concentrations were 0 (open square), 100 g/l (solid circle), 200 g/l (solid triangle), and 300 g/l (inverted solid triangle), respectively. The rabbit PrP was denatured in PBS buffer (pH 7.0) containing 2 M GdnHCl. The assays were carried out at 37°C.
(DOC)

Figure S2 **Macromolecular crowding inhibits aggregation formation of hen egg white lysozyme.** Aggregation of hen egg white lysozyme in the absence and in the presence of Ficoll 70, monitored by turbidity. The empirical Hill equation was fitted to the data and the solid lines represented the best fit. The final concentration of hen lysozyme was 350 µM. The crowding agent concentrations were 0 (open square), 100 g/l (solid circle), 200 g/l (solid triangle), and 300 g/l (inverted solid triangle), respectively. The assays were carried out at 37°C.
(DOC)
