# Supplementary material for: The Contrasting Effect of Macromolecular Crowding on Amyloid Fibril Formation
Source: PLoS One. 2012 Apr 30;7(4):e36288. doi: 10.1371/journal.pone.0036288 (PMC3340346; doi:10.1371/journal.pone.0036288)
Supplement: Supplementary file 1 [file pone.0036288.s001.doc]

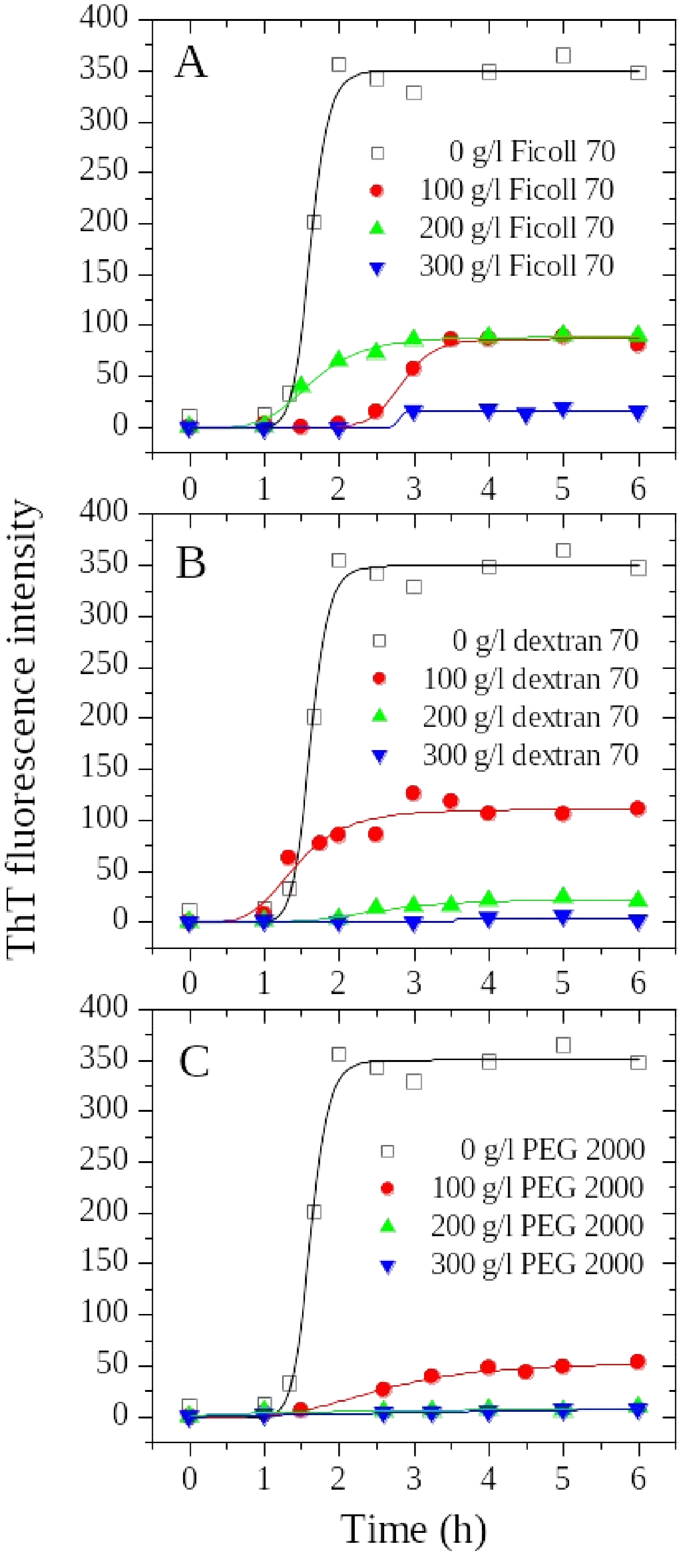

A
0 g/l Ficoll 70
100 g/l Ficoll 70
200 g/l Ficoll 70
300 g/l Ficoll 70
B
0 g/l dextran 70
100 g/l dextran 70
200 g/l dextran 70
300 g/l dextran 70
C
0 g/l PEG 2000
100 g/l PEG 2000
200 g/l PEG 2000
300 g/l PEG 2000
ThT fluorescence intensity
Time (h)
