# Supplementary material for: The Contrasting Effect of Macromolecular Crowding on Amyloid Fibril Formation
Source: PLoS One. 2012 Apr 30;7(4):e36288. doi: 10.1371/journal.pone.0036288 (PMC3340346; doi:10.1371/journal.pone.0036288)
Supplement: Supplementary file 2 [file pone.0036288.s002.doc]

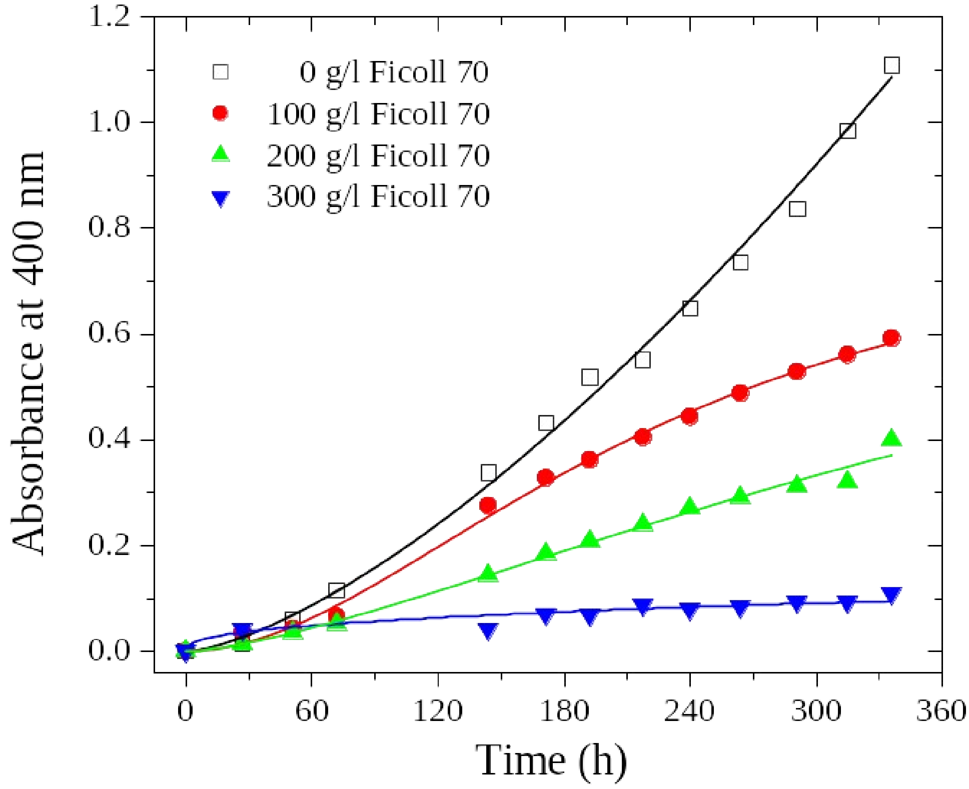

1.2
1.0
0.8
0.6
0.4
0.2
0.0
Absorbance at 400 nm
0 g/l Ficoll 70
100 g/l Ficoll 70
200 g/l Ficoll 70
300 g/l Ficoll 70
0
60
120
180
240
300
360
Time (h)
